# Sex-specific effects of small-sided games in basketball on psychometric and physiological markers during Ramadan intermittent fasting: a pilot study

**DOI:** 10.1186/s13102-021-00285-1

**Published:** 2021-05-23

**Authors:** Seifeddine Brini, Abderraouf Ben Abderrahman, Cain C. T. Clark, Sghaeir Zouita, Anthony C. Hackney, Karuppasamy Govindasamy, Urs Granacher, Hassane Zouhal

**Affiliations:** 1 Research Unit, Sportive Performance and Physical Rehabilitation, High Institute of Sports and Physical Education of Kef, University of Jendouba, Kef, Tunisia; 2 ISSEP Ksar-Essaid, University of La Manouba, Tunis, Tunisia; 3 Centre for Intelligent Healthcare, Coventry University, Coventry, CV1 5FB UK; 4 Department of Exercise & Sport Science, Department of Nutrition, University of North Carolina, Chapel Hill, NC USA; 5 Department of Physical Education & Sports Science, SRM Institute of Science and Technology, Kattankulathur, Tamilnadu India; 6 Division of Training and Movement Science, University of Potsdam, Potsdam, Germany; 7 M2S (Laboratoire Mouvement, Sport, Santé) - EA 1274, University of Rennes, F-35000 Rennes, France

**Keywords:** Team sports, Stress levels, Recovery, Performance, Fatigue

## Abstract

**Background:**

We assessed the effects of gender, in association with a four-week small-sided games (SSGs) training program, during Ramadan intermitting fasting (RIF) on changes in psychometric and physiological markers in professional male and female basketball players.

**Methods:**

Twenty-four professional basketball players from the first Tunisian (Tunisia) division participated in this study. The players were dichotomized by sex (males [G_M_ = 12]; females [G_F_ = 12]). Both groups completed a 4 weeks SSGs training program with 3 sessions per week. Psychometric (e.g., quality of sleep, fatigue, stress, and delayed onset of muscle soreness [DOMS]) and physiological parameters (e.g., heart rate frequency, blood lactate) were measured during the first week (baseline) and at the end of RIF (post-test).

**Results:**

Post hoc tests showed a significant increase in stress levels in both groups (G_M_ [− 81.11%; *p* < 0.001, *d* = 0.33, small]; G_F_ [− 36,53%; *p* = 0.001, *d* = 0.25, small]). Concerning physiological parameters, ANCOVA revealed significantly lower heart rates in favor of G_M_ at post-test (1.70%, *d* = 0.38, small, *p* = 0.002).

**Conclusions:**

Our results showed that SSGs training at the end of the RIF negatively impacted psychometric parameters of male and female basketball players. It can be concluded that there are sex-mediated effects of training during RIF in basketball players, and this should be considered by researchers and practitioners when programing training during RIF.

## Background

The demands of team sports in general, and basketball in particular, are determined by the interaction between physical, perceptual, mental, and technical-tactical aspects [1–3]. In this context, previous studies have suggested that small-sided games (SSGs) are a particularly relevant training strategy in basketball, since they can simultaneously promote the development of physical, physiological, and technical-tactical aspects required in competition [4–9].

However, studies involving cognitive effects of SSGs in basketball players are rare. To the authors’ knowledge, there are only two studies available in the literature so far [4, 10]. Moreover, previous investigations have demonstrated how increased mental demands can worsen perceived exertion [11, 12] and, importantly, physical [12, 13] and technical performance [11, 12] of team sport players. Furthermore, several studies have reported that psychometric disturbances affect sport performance [14, 15]. Thus, researchers have recommended using different scales to investigate recovery, perceived exertion, and well-being to monitor the physiological and psychometric status of players [8, 9, 16]. Indeed, these scales are effective in detecting early signs of tiredness and monitoring well-being to allow for optimization of training to achieve high-level performance [9, 16]. Previous studies have reported that the total quality recovery (TQR) scale is a valid tool that can be used to assess recovery state between training sessions [14, 17, 18]. In this context, Brink et al. [19] reported that TQR did not predict submaximal HR and was not associated with maximum shuttle race test performance. Moreover, Freitas et al. [17] reported that eleven days of an intense training load induced a significant decrease in TQR in volleyball players; whilst Osiecki et al. [18] showed that TQR was not related to the rating of perceived exertion (RPE), but was strongly associated with recovery state and biomarkers for muscle damage in professional soccer players. In addition, Selmi et al. [9] indicated that RPE in SSGs was not influenced by TQR or variability in well-being indices.

Of importance, training sessions, preparation phases, and basketball national and international tournaments can span the month of Ramadan intermitting fasting (RIF), which forces Muslim basketball players to fast, whilst concomitantly maintaining good performances and to continue training at the prescribed load or the intensity of their training [20–22]. Previous studies have revealed important physiological and cognitive RIF impacts in athletes, and basketball players in particular, because of the required abstention of food and water for a period exceeding 28 consecutive days [19–23]. In this context, Brini et al. [20] have shown for basketball and Baklouti et al. [23] for soccer that SSGs are common drills used by coaches for training during RIF, since SSGs allow maintenance and development of aerobic fitness in a practical way and lead to a greater motivation especially during RIF.

Moreover, male and female basketball players may respond differently to SSGs training during RIF for instance due to the menstrual cycle. In fact, females are obliged to interrupt their fasting state during the period of menstruation for religious reasons. Furthermore, there is a limited number of studies in the literature that examined the effects of sex during SSGs in basketball, where such studies have been limited to physiological effects in which male players induces a significant higher average heart rate and blood lactate concentration (during 4vs.4 SSG) in comparison with female players [24]. Moreover, research on the effects of sex on sport performances during RIF is scarce, and of the minimal available literature, results are unclear between sexes, perhaps owing to menstruation in females, which alters the observance of RIF during such days [25]. Thus, it would be interesting to control those parameters and to investigate the sex-mediated effects in association with SSGs training during RIF.

To the best of our knowledge, no previous study has investigated the effects of sex, in association with SSGs training, during RIF on psychometric and physiological markers in professional male and female basketball players. Accordingly, we sought to explore the sex-mediated effects of SSGs during RIF on well-being indices (e.g., including perceived ratings of sleep, fatigue, stress, and DOMS), TQR and physiological responses (e.g., heart rate frequency, blood lactate). Considering the previous literature [21, 23, 26], we hypothesized that: 1) psychometric and physiological parameters would be negatively affected by RIF in both sexes; and 2) it would be a sex-mediated effects in association with SSGs training during RIF.

## Methods

### Participants

Twenty-four professional basketball players from the first Tunisian division (Tunisia) participated in this study (Table 1). The players were assigned, according to their sex, to two groups (males [G_M_ = 12]; females [G_F_ = 12]), where each group included 3 guards, 3 shooting guards, 2 small forwards, 2 forwards, and 2 centers. The inclusion criteria for study participation were 1) participation in at least 90% of the training sessions; 2) Muslims who were fasting during Ramadan; and 3) having good health (no pain or injury reported); 4) and not taking any medications or other drugs; 5) for the female group, the menstrual cycle is longer than 28 days which starts at the beginning of Ramadan. The study was conducted during the competitive season and was approved by the Clinical Research Ethics Committee of the High Institute of Sports and Physical Education of Kef, University of Jendouba, Kef, Tunisia (approval No. 9/2018) and the protocol was conducted in accordance with the Declaration of Helsinki. All participants provided their written informed consent to participate in the study.

**Table 1:** Characteristics of the participating basketball players

Basketball players	Age (years)	Height (cm)	Mass (kg)	BMI (kg.m^**−2**^)
**G**_**M**_ **(*n*** **= 12)**	26.92 ± 2.97	1.94 ± 0.09	87 ± 6.95	23.15 ± 1.07
**G**_**F**_ **(*n =*** **12)**	24.75 ± 1.76	1.72 ± 0.08	71.50 ± 90.9	24.14 ± 1.48

Data are means and standard deviations. *G*_*M*_ male group, *G*_*F*_ female group, *BMI* Body mass index

### Study design and experimental procedure

Players were familiarized with all experimental procedures before the start of the study. To minimize any effects of diurnal variations, the training sessions were conducted at the same time of day. Players were instructed to wear the same footwear during all test sessions.

Overall, the study lasted 5 weeks and was conducted during the 2018/2019 in-season. The experimental period started with the beginning of the month of Ramadan in May 2019 and lasted until June 2019. During the Ramadan period, participants exercised five times per week between 5:30 and 7:00 pm and completed one game per week on the weekend. G_M_ and G_F_ completed a 4 weeks SSGs training (see details below) with a frequency of three sessions per week (Monday, Wednesday and Friday) over the course of the study period. No additional exercises or strength and conditioning sessions were conducted by any of the experimental groups.

Training sessions for both groups started with a 15-min warm-up program consisting of 5 min of low-intensity running, 5 min of dynamic stretching, and 5 min of skipping exercises [27], followed by technical and tactical drills based on basic basketball movements (i.e., offensive, ready stance, running, change of direction, linear sprint, stopping, pivoting, and jumping exercises), specific basketball movements (triple threat position, pivot, face up or one- and two-phase stop), basketball technique fundamentals (dribbling, passing, and shooting), basic defensive movements (defensive stance, defensive slide, denial defense, and box-out) and a simulated game at the end of every session [27], which were identical for both groups. Both groups completed the same training volume (~ 90 min per session) over the course of the study (Table 2).

**Table 2:** Weekly training program during the month of Ramadan for male and female players

Days	Weekly training program during the month of Ramadan for males and females
**Monday**	-Warm up, 15 min -Small-sided games, (3 × 4 min),2 min recovery between bouts -Free throw shooting, 10 min -Technical training, 20 min -Tactical training, 20 min
**Tuesday**	-Warm up, 15 min -Specific basketball fundamental training, 15 min -Moderate intensity mid-range and 3 point shot exercises, 20 min - Technical/Tactical training, 40 min
**Wednesday**	-Warm up, 15 min -Small-sided games, (3 × 4 min),2 min recovery between bouts -Free throw shooting, 10 min -Technical training, 20 min -Tactical training, 20 min
**Thursday**	-Warm up, 15 min -Specific basketball fundamental training, 15 min -Moderate intensity ball drill transition training, 16 min -Moderate intensity mid-range and 3 point shot exercises, 20 min -Tactical training, 20 min
**Friday**	-Warm up, 15 min Small-sided games, (3 × 4 min),2 min recovery between bouts -Free throw shooting, 15 min -Low intensity 3 point shooting exercises, 25 min
**Saturday**	-Match
**Sunday**	-Recovery

### Small-sided games training program

SSGs were performed on half-court (14 × 15 m) (35 m^2^ per player) at the beginning of the training session. The duration of each SSG was 12 min. The defensive strategy allowed was player-to-player only. The training regimes consisted of three 4-min bouts interspersed by 2 min of passive recovery, the court size and the duration of 3 vs. 3 SSGs were strictly controlled, as reported in previous studies [12, 28]. The players were asked to perform at maximum effort during the games.

Two coaches were positioned around the perimeter of the court to encourage the players and to provide new balls when necessary to allow for continuous play and to maintain the game pace during the SSGs sessions (Table 3).

**Table 3:** Rules of the small-sided game in basketball

New ball possession	Scoring, foul, rebound (offensive or defensive), steal, turnover, out of bounds
**Ball clearance**	Yes- pass to assistant (except after offensive rebound)
**Shot clock**	12 s
**Free throw**	No – 1 point to offensive team
**Referees**	2
**Score**	Yes
**Encouragement**	Yes

### Training load monitoring

To determine whether the players’ global training load remained constant throughout the study, the session rating of perceived exertion (RPE) training score was taken after each session. About 30-min after training, players were asked to rate the global intensity of the entire workout session using the category ratio-10 RPE scale according to the methods described by Foster [29]. A daily training load was created by multiplying the training duration (minutes) by the session RPE. The weekly training load was determined by summing the daily training loads for each athlete during each week.

### Anthropometrics and fitness measurements

Body mass (kg) was measured with an electronic scale (Pharo 200 Analytic, Germany) and height (m) with a portable stadiometer (Seca, Maresten, UK). Maximal oxygen consumption (VO_2max_) was estimated using the 20-m shuttle run test, according to the Léger and Gadoury equation [30].

### Psychometric markers

Fifteen minutes before the warm-up, each player was asked to complete ratings of well-being indices (quality of sleep, fatigue, stress, and DOMS) considering the timeline from the last SSGs training session until the moment of the new training session. Players rated each index using a scale from 1 to 7 points, where 1 indicated “very, very low” (fatigue, stress, and DOMS) or “good” (quality of sleep) and 7 indicated “very, very high” (fatigue, stress, and DOMS) or “bad” (quality of sleep) [8]. The sum of these 4 scores was used to calculate the HI. A higher HI score indicates a more negative state of well-being. After each player completed the well-being indices, recovery state was evaluated using the TQR scale [9]. The TQR scale ranges from 6 to 20, where 6 indicates “very, very poor recovery” and 20 indicates “very, very good recovery.” This scale has been used in previous studies as an indicator of athletes’ perceived recovery [9]. Immediately at the end of each SSG, RPE was evaluated using the Borg CR-10 scale [29] to assess subjective training intensity.

### Physiological measures

HR was continuously monitored throughout the training intervention by HR monitors (Polar Team Sport System; Polar-Electro OY, Kempele, Finland) and was recorded at 5-s intervals. To reduce HR recording error, all players were regularly asked to check their HR monitors during SSGs. The mean HR for each bout was calculated from continuous HR data. The resulting mean HRs for each SSG were further averaged to obtain the overall mean HR (HR_mean_).

Blood samples for the determination of blood lactate concentration ([Lac]) were collected 3 min after training in the absence of an active recovery [31]. Blood samples, taken from the fingertip of the index finger, were analyzed by a validated portable analyzer (Lactate Pro, Arkray, Japan) [31].

### Statistical analyses

All the data were expressed as Mean ± SD. The Shapiro Wilk test identified all variables as normally distributed. Baseline between group differences were computed using t-tests for independent samples. If statistically significant between group baseline differences were found, analysis of covariance (ANCOVA) with group as between-subject comparator (G_M_ and G_F_) was performed, and baseline data as a covariate [32]. In addition, group-specific repeated-measures analyses of variance (time: pre, post) were applied to evaluate within-group pre-to-post performance. Effect sizes (ESs) were calculated by converting partial eta-squared to Cohen’s *d*. Effect sizes were classified as small (0 ≤ *d* ≤ 0.50), medium (0.50 ≤ *d* ≤ 0.80), and large (d ≥ 0.80) [33]. The significance level was set at *p* ≤ 0.05, a priori. All analyses were performed using Statistical Package for Social Sciences (SPSS) version 20.0 (SPSS, Inc., Chicago, IL).

## Results

All players from both groups completed the study, according to the previously described methodology. Training load remained constant throughout the study, and no injuries were reported. During the four weeks intervention period, adherence rates were 96.5% for G_M_ and 95.8% for G_F_. The average playing time per game was 29.4 ± 1.8 min for G_M_ and 29.2 ± 1.5 min for G_F_. No statistically significant between group differences were observed for these measures.

ANCOVA was carried out for psychometric and physiological markers, because there was a significant difference for baseline values between the two groups, except for stress levels, TQR and Lac.

### Psychometric markers

Table 4 presents baseline data for psychometric markers. ANCOVA revealed no significant effect of group for any measure of psychometric markers at post-test (sleep [*d* = 0.005, small, *p* = 0.742]; fatigue [*d* = 0.019, small, *p* = 0.526]; DOMS [*d* = 0.011, small, *p* = 0.636]; HI [*d* = 0.013, *p* = 0.606]; RPE [*d* = 0.029, small, *p* = 0.438]). However, significant pre-to-post-test increases were found for those parameters in both groups, except for RPE where there was a pre-to-post-test increase for G_M_, only (sleep: G_M_ [*d* = 0.93, large, *p* < 0.001] and G_F_ [*d* = 0.86, large, *p* < 0.001]; fatigue: G_M_ [*d* = 0.89, large, *p* < 0.001] and G_F_ [*d* = 0.71, *p* < 0.001]; DOMS: G_M_ [*d* = 0.90, large, *p* < 0.001] and G_F_ [*d* = 0.86, large, *p* < 0.001]; HI: G_M_ [*d* = 0.95, large, *p* < 0.001] and G_F_ [*d* = 0.90, large, *p* < 0.001]; RPE: G_M_ [*d* = 0.72, medium, *p* < 0.001] and G_F_ [*d* = 0.28, small, *p* = 0.061]).

**Table 4:** Baseline data, baseline-adjusted post-test data, as well as pre-to-post changes of psychometric and physiological markers for the male group (G_M_; *n* = 12) and the female group (G_F_; *n* = 12)

Variables	Baseline				Post-test ^**₤**^					Pre-to-post changes			
	G_**M**_	G_**F**_	%↑	***p***	G_**M**_	G_**F**_	Group effect			G_**M**_		G_**F**_	
	Mean ± SD (95%IC)	Mean ± SD (95%IC)			Mean ± SD (95%IC)	Mean ± SD (95%IC)	***d***	***p***	%↑	***d***	***p***	***d***	***p***
**Sleep**	3.58 ± 0.673 (3.15–4.01)	4.33 ± 0.53 (3.99–4.66)	20.95	0.006	6.21 ± 0.17 (5.84–6.57)	6.12 ± 0.17 (5.75–6.48)	0.005	0.742	−1.45	0.93	< 0.001	0.86	< 0.001
**Fatigue**	3.16 ± 0.96 (2.55–3.77)	4.41 ± 0.57 (4.05–4.78)	39.56	0.001	5.62 ± 0.24 (5.11–6.13)	5.87 ± 0.24 (5.36–6.38)	0.019	0.526	4.45	0.89	< 0.001	0.71	< 0.001
**DOMS**	1.91 ± 0.74 (1.44–2.39)	2.61 ± 0.60 (2.23–2.99)	36.65	0.019	4.85 ± 0.17 (4.49–5.22)	4.72 ± 0.17 (4.36–5.09)	0.011	0.636	−2.68	0.90	< 0.001	0.86	< 0.001
**HI**	12.10 ± 2.07 (10.78–13.41)	14.91 ± 1.88 (13.71–16.11)	23.22	0.002	22.14 ± 0.46 (21.17–23.10)	22.52 ± 0.46 (21.56–23.48)	0.013	0.602	1.72	0.95	< 0.001	0.90	< 0.001
**RPE**	5.83 ± 0.89 (5.26–6.39)	6.97 ± 0.81 (6.45–7.49)	19.55	0.003	7.34 ± 0.18 (6.97–7.72)	7.56 ± 0.18 (7.19–7.94)	0.029	0.438	2.99	0.72	< 0.001	0.28	0.061
**HR (beat/min)**	184.50 ± 1.32 (183.65–185.34)	187 ± 1.66 (185.94–188.05)	1.36	0.001	180.26 ± 0.53 (179.14–181.37)	183.32 ± 0.53 (182.21–184.43)	0.381	0.002	1.70	0.67	< 0.001	0.82	< 0.001

Data are means and standard deviations. *G*_*M*_ male group, *G*_*F*_ female group, *DOMS* muscle soreness, *HI* Hooper indices, *HR *heart rate, *RPE* rating of perceived exertion

*d*: effect size Cohen’s d

↑: Value Female training group × 100/value Male training group - 100

^₤^: Baseline-adjusted post-test data

Concerning stress levels and TQR, ANOVA revealed a significant main effect of time ([*p* < 0.001, *d* = 0.82, large]; [*p* < 0.001, *d* = 0.90, large] respectively) and a significant group x time interaction only for stress levels (*p* = 0.014, *d* = 0.44, small). Bonferroni corrected post hoc tests showed a significant increase in stress levels for both groups (G_M_ [− 81.11%; *p* < 0.001, *d* = 0.33, small]; G_F_ [− 36.53%; *p* = 0.001, *d* = 0.25, small]).

### Physiological measures

**Fig. 1:**
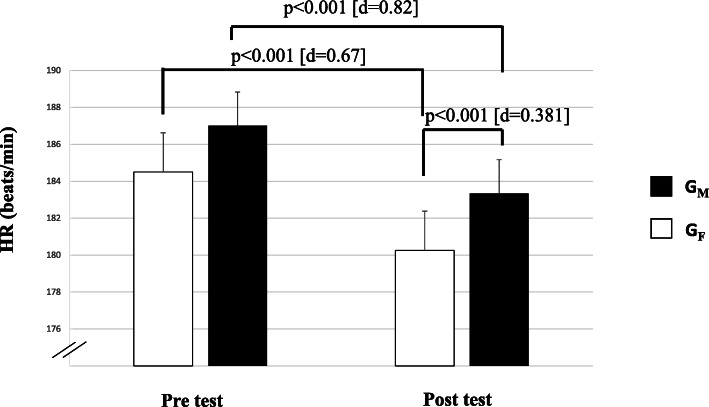
Variation of heart rates mean during Ramadan for male, female groups. Legend. HR: heart rates; GM: male group; GF: female group

Table 4 presents baseline data for physiological markers. ANCOVA revealed significantly lower HR in favor of males at post-test (1.70%, *d* = 0.38, small, *p* = 0.002). Moreover, significant pre-to-post-test changes for HR were found for both groups (G_M_ [2.30%, *d* = 0.67, medium, *p* < 0.001] and G_F_ [1.96%, *d* = 0.82, large, *p* < 0.001]) (Fig. 1). Concerning Lac, ANOVA revealed only a significant main effect of group (*p* = 0.031, *d* = 0.36, small).

## Discussion

This study investigated the sex-mediated effects of SSGs training during Ramadan on psychometric status (quality of recovery and well-being indices) and physiological responses (HR, blood lactate) in professional male and female basketball players. The main findings of our study indicated that psychometric parameters (i.e., well-being indices) were negatively impacted as a result of RIF for both groups, and that SSGs training elicited a higher HR for female professional basketball players, at the end of RIF. Additionally, players maintained a high training load during the period of the study (2000 AU for G_M_ and 1800 AU for G_F_) in order to sustain competitive fitness and muscle mass. Our values were similar to these reported in Italian soccer players (1900 AU) (in-season) but lower than overreached rugby league players [34].

Blood lactate concentration did not significantly change throughout RIF for both groups. These results indicated that the participating players maintained similar metabolic conditions for each SSGs session. Moreover, no significant sex-mediated effects were found, which could be explained by the fact that blood lactate concentration is affected not only by the lactate diffusion into the blood but also by the blood volume into which it diffuses; indeed, because women have a smaller total blood volume (4.0 to 4.5 l) than men (5.0 to 6.0 l), a smaller amount of lactate would be required to produce a similar blood lactate concentration [35].

We found that HR significantly decreased at the end of Ramadan for both groups in comparison with the first week. Accordingly, Al Suwaidi et al. [36] explained this decrease by the association of fasting with catecholamine inhibition and reduced venous return, causing a decrease in the sympathetic tone, which leads to a decrease in blood pressure, HR, and cardiac output. Moreover, our results showed that the male group had a significantly lower HR in comparison with the female group. These values could be explained by the recovery capacity of our participants between bouts. In fact, it has been reported that male players have a quicker recovery ability than the female players. Sex-related differences might also be a result of the males’ having a 5–30% greater VO_2_max and having lower body fat percentage than their female counterparts [37].

Concerning RPE scores, we recorded a higher value at the end of RIF. Our findings could be explained by the increased muscle fatigue during the RIF. This increase is concordant with previous research that reported an increased sensation of fatigue during RIF [26]. Indeed, decrements in physical function can lead to an increase in perceived exertion, an earlier onset of fatigue, and, thus, an increased risk of injury or illness [38].

In this study, psychometric markers, such as stress levels, DOMS, HI, TQR, were negatively affected at the fourth week in comparison with the beginning of RIF for both groups. These results could putatively be explained by the changes in the daily habits of players during Ramadan [35]. In fact, players tend to prepare for the period of fasting during Ramadan by rising earlier and eating a meal before sunrise [35]. Resultantly, players tend to be impacted by partial sleep deprivation, which can affect the higher cognitive centers of the central nervous system which negatively affect mental and athletic performances [35, 39, 40]. Furthermore, the reduction in mental activity has been posited as one of the main reasons for performance decrements [12, 26]. In addition, fatigue has been shown to reduce muscle glycogen depletion and/or alter neurotransmitter activity that could, in turn, negatively influence cognition and motor skill performance [41].

### Limitations

Although we present a novel addition to the literature, our study has some limitations that warrant consideration. Firstly, the small sample size, which reduces our statistical power; however, this study merely presents preliminary work, and was not designed to be powered as a randomized controlled trial. Secondly, time-motion variables were not included (i.e., distance covered, sprint number, and run at high intensity), which would have provided a further level of fidelity to our study. Third, only a 3 vs. 3 SSG format was used; thus, changing duration, pitch size, recovery period, number of players, etc., may provide further information or yield differing responses. Finally, the period within the year when Ramadan occurred must be considered; indeed, future studies may have different results by investigating the month of Ramadan under different climatic circumstances.

## Conclusions

Our results showed that SSGs training, at the end of RIF, negatively affected psychometric parameters of males and females, in particular in HI, which indicates a more negative state of well-being. Moreover, a significantly higher HR recorded in favor of female professional basketball players. Thus, it can be concluded that there are sex-mediated effects of training during RIF in professional basketball players, and this information should be considered by both researchers and practitioners when programing training during RIF.

### Practical applications

- Higher stress levels, fatigue, and DOMS, were recorded at the end of RIF, which may negatively affect technical aspects and physical performance in basketball.
- Total quality of recovery decreased by the end of Ramadan which was accompanied by a decrease on sleep quality.
- Provision of a sufficient recovery during the RIF is recommended to help avoid performance deterioration and overtraining.

## Data Availability

The datasets generated during and analyzed during the current study are not publicly available due to confidential information about the participants but are available from the corresponding author on reasonable request.

## Abbreviations

DOMS: Delayed onset of muscle soreness
TQR: Total quality recovery
RIF: Ramadan intermitting fasting
ANCOVA: Analysis of covariance
RPE: Rating of perceived exertion
HI: Hooper index
HR: Heart rate
Lac: Lactate concentration
BMI: Body mass index
